# A Literature Review of Metagenomics and Culturomics of the Peri-implant Microbiome: Current Evidence and Future Perspectives

**DOI:** 10.3390/ma12183010

**Published:** 2019-09-17

**Authors:** Leonardo Martellacci, Gianluca Quaranta, Romeo Patini, Gaetano Isola, Patrizia Gallenzi, Luca Masucci

**Affiliations:** 1 Fondazione Policlinico Universitario A. Gemelli IRCCS, Institute of Dentistry and Maxillofacial Surgery, Università Cattolica del Sacro Cuore, 00168 Rome, Italy; lmartellacci@virgilio.it (L.M.); patrizia.gallenzi@unicatt.it (P.G.); 2 Fondazione Policlinico Universitario A. Gemelli IRCCS, Institute of Microbiology and Virology, Università Cattolica del Sacro Cuore, 00168 Rome, Italy; gquaranta88@gmail.com (G.Q.); luca.masucci@unicatt.it (L.M.); 3 Department of General Surgery and Surgical-Medical Specialties, School of Dentistry, University of Catania, 95124 Catania, Italy; gaetano.isola@unict.it

**Keywords:** implantology, microbiology, periodontal medicine, metagenomics, culturomics, plaque control

## Abstract

**Background and objectives:** In recent years, many different culture-independent molecular techniques have been developed with the aim of investigating the not yet cultivated part of the resident flora of the oral cavity and of analyzing the peri-implant and periodontal flora both in healthy and diseased sites. The most used technologies are Roche 454 pyrosequencing, Illumina HiSeq/MiSeq, ABI SOLiD and Ion Torrent. Due to these methods, two different approaches are available: Metagenomics and the 16S gene analysis. A complementary strategy was also recently developed: Culturomics. Culturomics consists of different culture conditions that allow a very rapid bacterial identification. The focused question of this review was developed in PICO format in order to investigate the role of metagenomics, 16S gene analysis and culturomics (interventions) in the differential study (comparison) of the peri-implant and periodontal microbiome (outcome) in humans (participants). The secondary aim was the characterization of currents limits and future applications of the three techniques. **Methods**: The authors performed a literature search on three databases (Web of Science, Scopus and PubMed) from 01/01/2003 to 31/06/2019. Date of last search was: 25/08/19. Any type of article dealing with the analysis of periodontal and peri-implant flora with metagenomic, culturomic or 16S gene analysis was included. No language restrictions were applied. Risk of bias for RCT was assessed using the Cochrane collaboration’s tool whereas case-control and cohort studies were evaluated through the Newcastle–Ottawa scale. **Results**: The initial search resulted in 330 titles in total. After careful evaluation of all results no studies were found to satisfy the primary outcome of the present review. Hence a narrative review dealing with the secondary aim was performed. **Conclusions**: Metagenomic and 16S gene analysis approaches contributed in clarifying some crucial aspects of the oral microbiome. Based on the reported evidence some bacteria could be found around teeth and implants even in the absence of signs of inflammation and other species are more frequently found in supragingival peri-implant biofilm. Teeth and implants (even if adjacent) seem not to share the same microbiome and healthy teeth have a more diversified one. The same analyses also highlighted that the oral biofilm of smokers is composed by more periodontopathogen bacteria compared to non-smokers and that geographical location and ethnicity seem to play a role in bacterial composition. Culturomics, which has not yet been applied to the study of oral microbiota, consists of the use of different culture conditions and of the identification by matrix-assisted laser desorption ionization–time of flight mass spectrometry (MALDI–TOF MS) with the aim of increasing the bacterial repertoire and avoiding the limits of molecular methods. In order to better evaluate perspectives and limits of the all presented approaches further studies comparing the different molecular techniques are encouraged. This review received no funding.

## 1. Introduction

The term “microbiome” has been used for referring to the genomes of all microbes living inside and on the human body [1]. In literature, frequently, the term “microbiome” is confused with microbiota, which is the set of bacterial communities that reside in an environment and that constitute the microbiome. In 2016, over 700 prokaryotic taxa were isolated in the oral cavity, of which 54% were represented by named species; 14% were non-named but cultivated species; of the remaining 32% only their phylotype was known. The same analysis evidenced that each individual would host approximately 300 species in its mouth [2]. Part of these estimates comes from bacterial cultures, and part from culture-independent bacterial identification methods, like the 16S rRNA gene analysis [3,4,5]. The HOMD (Human Oral Microbiome Database) provides information on these bacteria that can be found into the oral cavity. The HOMD is a database of 16S rRNAs, which also proposes a scheme for naming species or phylotypes that have not been named to date. It allows phenotypic, phylogenetic, clinical and bibliographic information about the bacteria present in the catalog. As part of the HOMD, the Human Microbiome Project (HMP) provides genomic sequences for around 400 oral bacterial taxa that account for the 58% of known species [6].

The most used technologies for the identification of the not yet cultivated part of the resident flora of the oral cavity are: Roche 454 pyrosequencing, Illumina HiSeq/MiSeq, ABI SOLiD and Ion Torrent. With the developing of these methods, two different approaches became available: Metagenomics and the 16S gene analysis. A complementary strategy was also recently developed: Culturomics. It consists of multiple culture conditions combined with the rapid identification of bacteria and allows the culture of hundreds of new microorganisms providing exciting new perspectives on host–bacteria relationships.

The primary aim of this review (provided in PICO format) was to investigate the role of metagenomics, 16S gene analysis and culturomics in the differential study of the peri-implant and periodontal microbiome in humans. The secondary aim was the characterization of currents limits and future applications of the three techniques.

## 2. Materials and Methods 

Two authors (LM and RP) performed a systematic literature search in PubMed, Web of Science and Scopus databases. The analysis was conducted with the aim of identifying any coherent article published between 1 January2003 and 31 June 2019. The time interval was chosen since no literature reports regarding any of the three analyzed techniques before 2003 were available. No language restrictions were applied. Risk of bias for RCT was assessed using the Cochrane collaboration’s tool whereas case-control and cohort studies were evaluated through the Newcastle–Ottawa scale.

A combination of the sequent free text words has been used: “Metagenomics”, “culturomics”, “16S gene analysis”, “periodontal”, “peri-implant”, “oral cavity” and “mouth”. The free text words have been variably connected among them with the Boolean operators “AND” and “OR”.

Studies have been screened independently and in duplicate by two calibrated authors (LM and RP). In case of disagreement the two authors discussed with one of the authors’ supervisors (L Mas or PG) until consensus. The reviewers screened all the titles and abstracts and for studies apparently coherent with the review topic they analyzed the full-text. All the analyses were made with the help of a data extraction form.

## 3. Results 

**Figure 1:**
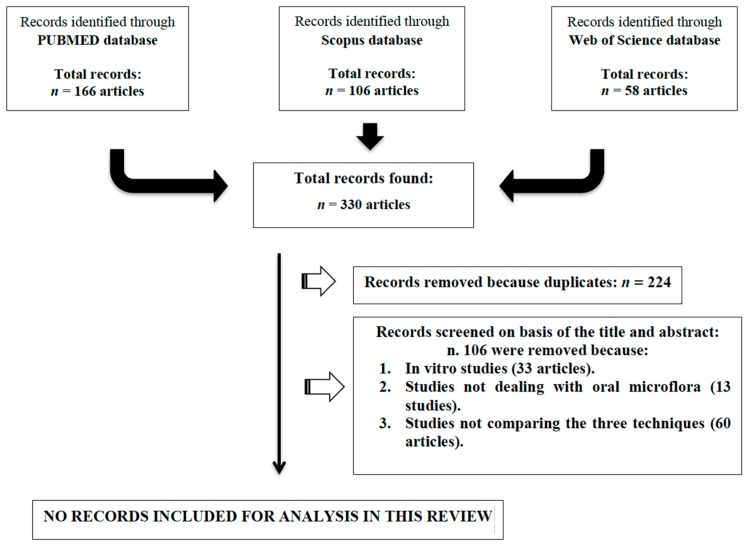
Flow chart of the search strategy.

As reported in Figure 1 the electronic search resulted in 330 items but no articles gave substantial information regarding the primary aim of the review. Hence the authors decided to perform a narrative synthesis of the existing literature in order to pursue the secondary aim of the review.

## 4. Literature Review 

### 4.1. Metagenomics

In recent years, many different culture-independent molecular techniques have been developed with the aim of investigating, even with certain limits, the part of the resident flora of the oral cavity not yet cultivated. A high number of microorganisms can be analyzed through the use of the next generation sequence (NGS) technologies without bacterial culture. The most used NGS technologies in investigating human oral diseases are Roche 454 pyrosequencing, Illumina HiSeq/MiSeq, ABI SOLiD and Ion Torrent semiconductor sequencers [7]. These new techniques allow two different approaches of analysis. The first is Shotgun metagenomics, which "fragments" and then "reads" the entire DNA in the sample, detecting viruses, bacteria and parasites. The second approach has a defined target, and the sequence that is usually investigated with this method is the 16s rRNA, a fundamental part of the set of prokaryotic functional genes that is only slightly affected by horizontal transfer [8].

The word “metagenomics” was introduced by Jo Handelsman et al. in 1998 [9]. In 2005, the term “metagenomics” was defined by Chen and Pachter as “the application of modern genomics techniques to the study of communities of microbial organisms directly in their natural environments, bypassing the need for isolation and lab cultivation of individual species” [10]. It is a type of analysis that was developed in order to study microorganisms by sequencing their DNA. Xu and Gunsolley described the differences between metagenomics and 16S DNA sequencing: Metagenomic approach needs less amount of PCR amplification than 16S DNA sequence analysis and is able to quantify individual bacterial species in samples. Moreover, contaminating human sequences are removed. Another important aspect is that it is possible to identify genetic segments potentially associated with health or disease. 16S DNA has lower costs and less computational requirements than metagenomics, for these reasons it is often preferred by researchers. Nevertheless in the study of the oral microbiome, metagenomics has been demonstrated to provide more detailed information [7].

The other approach aside from metagenomics is 16S rRNA analysis. This gene has some hypervariable regions that could be considered as the genetic fingerprint of a single microorganism. Such regions have been numbered from V1 to V9 and for every one of them specific primers are produced. Some authors pointed out that there is no hypervariable region that alone allows the diagnosis of all the bacterial genera examined, so it would be advisable to investigate different regions [11,12]. One example is found in the *Synergistetes* spp. that is not identifiable by the primer for V5–V6 regions but, if searched with the primer V1–V2, they represent more than 1% of the classes. *Fusobacteria* spp. undergoes a similar outcome and *Clostridia* spp., on the other hand, is four times more abundant in the samples if analyzed with V5–V6 [12].

### 4.2. The Peri-Implant and Periodontal Microbiome

In literature, there are numerous studies that aim to analyze the peri-implant bacterial flora both in healthy sites and those affected by various forms of disease (mucositis and peri-implantitis) and to make a comparison with the flora present around the dental elements (both healthy ones and those with varying degrees of periodontal disease).

Quirynen and Elter reported that the bacterial flora around implants was mostly composed of *Streptococci* (45%–86%), *Actinomyces naeslundii*, *Actinomyces oris*, *Actinomyces meyeri*, *Neisseria* spp. and *Rothia* spp. [13,14]. These two studies used different investigation techniques: Elter analyzed the bacterial biofilm grown on the healing abutment using the scanning electron microscopy, the second electron and the Rutherford back-scattering detection method. Nevertheless these methods were limited by not being able to clearly identify the sub-gingival biofilm. Quirynen instead used the DNA–DNA checkerboard hybridization technique [13,14].

According to studies by Persson et al. and Casado et al. and to the systematic review by Patini et al., sometimes bacteria associated with periodontitis including *Fusobacterium nucleatum*, *Prevotella intermedia*, *Porphyromonas gingivalis*, *Aggregatibacter actinomycetemcomitans* and *Filifactor alocis* are found around the implants, even in the absence of obvious signs of inflammation [15,16,17]. The presence of such bacterial species in periodontal and peri-implant sulcus has also been linked with an increasing number of diseases affecting other organs beyond the mouth [18,19,20,21,22,23,24]. Persson et al. used the DNA–DNA checkerboard hybridization technique [15] while Casado et al. the analysis of the crevicular fluid instead [16].

Zaura et al. adopted the Roche 454 platform to pyrosequence the V5–V6 variable region with the aim of investigating the composition of oral microbiome in health condition. They collected samples of stimulated saliva from three healthy subjects and then, through sterile microbrushes, collected samples on the mucous surfaces and on different tooth surfaces. The result was that all three microbiomes analyzed shared 47% of their composition, that is 387 of the 818 OTU (operational taxonomic unit) found. These phylotypes together contributed 90%–93% to the formation of each microbiome. Fifty-one of these shared OTUs had high concentration (>0.1% of the microbiome) and together they accounted for the 62%–73% of the individual microbiome. At a higher taxonomic level, 72% of all taxa were shared by all three microbiomes, contributing 99.8% of all reads. Only 2%–11% were specific individuals. From these evidences Zaura et al. proposed the concept of a healthy core microbiome, site-specific between saliva and mucous membranes and between teeth and saliva [25].

Dabdoub et al. collected samples of subgingival plaque using sterile endodontic paper points (*n* = 10, DENTSPLY) placed in the gingival and peri-implant sulcus for 10 s. The V1–V3 and V7–V9 regions of the 16S were used as primers for pyrosequencing in order to try to understand if the teeth share the microbiota with the adjacent dental implants. From the results obtained in this study, it was found that teeth and implants have two different microbiota, as dental implants create a real microenvironment that selects the bacterial flora. In fact, the same authors found that around 85% of the study participants shared less than 8% of the species between the tooth and implant if bacterial species were analyzed with an abundance greater than 1% in the two micro-ecosystems. Another finding from this study showed that the microbiota was more variegated around teeth rather than around implants, especially in health conditions [26].

Three years later, in another study, Dabdoub et al. examined 25 subjects with chronic periodontal disease and 25 healthy patients. The subgingival microbiome was studied through WGS (whole genomic shotgun) and the samples were recovered with sterile endodontic paper points. Unlike the other types of analysis, the shotgun allows the identification not only of bacterial DNA, but also of that belonging to viruses, fungi and archaea. This sampling method combined with WGS revealed that between 62.96% and 77.22% of the entire DNA obtained did not belong to the oral microflora. In this study, the definition of the core microbiome was “the one present in at least 80% of the subjects”. Of the members of the microbial community 75% belonged to 46 species of the genera *Streptococcus*, *Veillonella*, *Actinomices*, *Corynebacterium*, *Neisseria*, *Fusobacterium* and *Selenomonas* and 22% belonged to the core microbiota. Study results revealed that viruses and archaea are not part of the core, whereas fungi are represented by *Candida albicans*, present in 87% of the subjects studied [27].

Koyanagi et al. aimed to investigate the differences in the microbiota of teeth affected by periodontal disease, implants in peri-implant disease and clinically healthy dental implants. In this study the Roche 454 platform was chosen and the primers used were the 27F (region V1) and the 1492R. The samples were recovered using endodontic paper points placed in the deepest periodontal or peri-implant pocket and removed after 30 s. The authors of this study hypothesize that the surface roughness and free energy (wettability) of the implants may have a crucial role in the selection of the bacteria that adhere to the biofilm on the surface of the implant [28]. The results of this study described the bacterial flora in cases of peri-implantitis, neglected the description of the flora on an implant showing no signs of inflammation.

To better understand the variations in the subgingival microbial flora, Shchipkova et al. selected 15 smoking patients and 15 non-smokers, with a moderate to severe degree of periodontal disease. Plaque samples were collected in pockets deeper than 5 mm using endodontic paper points, held in place for 10 s. The authors used A17 and 317 primers (Biosynthesis, Lewisville, TX, USA). At the end of the cloning procedures, the products were purified with the Millipore kit. The obtained results highlighted no difference in the number of species not yet cultivated between the two groups examined that are respectively 38.8% for smokers and 44.5% for non-smokers. The taxa not yet cultivated were composed mainly for both groups from *Synergistes*, *Lachnospira*, *Desulfobulbus*, *Selenomonas*, *Neisseria*, *Veillonella*, *Eubacterium* and *Catonella*. Smokers had higher quantities of *Parvimonas*, *Campylobacter*, *Treponema*, *Bacteroides* and *Fusobacterium* while non-smokers had higher quantities of *Streptococcus*, *Veillonella* and *Neisseria*. Significant differences were found in the prevalence of some species between the two groups: *Parvimonas micra* and *Campylobacter gracilis* represented the largest sub-gingival flora in smokers, while non-smokers had higher levels of *Veillonella* spp., *Streptococcus sanguinis* and *Tannerella forsythia*. Although in this study the clinical manifestations of periodontal pathology were completely similar in the two groups investigated, it was found that there are differences in the sub-gingival flora between smokers and non-smokers. Indeed, it appears from the data of this study that the biofilm associated with smoking patients is richer in periodontopathogens bacteria. It is plausible to hypothesize that there is a difference in the bacterial flora between the two microenvironments due to the habit of smoking even in subjects that do not show signs of periodontal pathology [29].

Regarding the composition of the oral microbiome, most studies in the literature cite HMP. HMP described such composition by taking samples from seven oral and two oropharyngeal sites from 242 healthy subjects [30]. Thus, it was found that 95% of the entire oral microbiome is composed of *Firmicutes*, *Bacteroidetes*, *Proteobacteria*, *Fusobacteria* and *Actinobacteria* [11,31]. Of all the body sites examined, the oral cavity has the greatest α diversity (species richness) after the intestine; if instead the same sites are compared between different subjects (β diversity), the oral sites have less variability compared to other body sites [31,32]. At the sub-gingival level, there is significant variability among individuals, especially at levels lower than the genus, in the relative abundance of the OTUs [31]. Although the HMP proposes an important volume of data, the 16s rRNA datasets are mostly composed of samples from US medical students and their data are not available, so it is not possible to evaluate the homogeneity of the samples and any ethnic or geographical differences [11].

Nasidze et al. conducted a study in which a total of 120 subjects were enrolled, divided into groups of 10 individuals from six different regions (each region provided two groups): South America, North America, Africa, Europe (Germany and Poland), the Middle East and Asia. Analyzing the 16S of the salivary microbiome, the authors found differences between the various geographical areas, even though 70% of all the sequences were common among the various subjects. However, the MDS (multi-dimensional scaling) analysis carried out in this study would suggest that geography would not significantly affect the bacterial composition [33]. The great limitation of this study was the reduced number of subjects, the lack of consideration for some variables and the variety of subjects to enroll in the study. Environmental and cultural (and therefore food) differences were not taken into account. Age, sex and oral status of the subjects were not reported.

To assess if ethnic differences also lead to variation in the oral microbiome composition, Mason et al. analyzed the dental plaque of 192 subjects belonging to four ethnic groups (non-Hispanic black, non-Hispanic white, Hispanic and Chinese) finding some differences [34] in agreement with the previous literature [11,35,36,37]. Genetic rather than environmental differences seem to exert a selection on the bacterial flora because two ethnic groups, while sharing the same lifestyle and the same eating habits showed substantial differences in the microbiome [34].

A longitudinal study of 85 patients observed weekly for 3 months showed a certain degree of variability in the composition of the lingual microbiome and in that of other body niches. Such evidence could lead to the theory that the composition of a microbiome and its variability over time are individual characteristics [38]. The same theory was strengthened by Xu et al. that affirmed that the phases of dentition as well as aging select the bacterial composition of the oral cavity [39].

Zheng et al. compared the results obtained from the metagenomic analysis with Illumina (Miseq) of the hypervariable region V1–V3 with those obtained from the V3–V4 region (the most used in pyrosequencing protocols). The comparison was made on the basis of taxonomic richness (α diversity) and species uniformity (β diversity) found at the sub-gingival level in diabetic and non-diabetic patients affected by periodontitis. The β diversity was found to be similar for the two hypervariable regions, while for α diversity, it was found that the V1–V3 region provides a greater richness of phylotypes [40].

Szafranski et al. conducted a pilot study to compare the taxonomic resolution power of two different 16s rRNA primers: The one complementary to the V1–V2 region and the one for the V5–V6. The chosen NGS platform was Illumina (Miseq). The population of the study was chosen in a random manner; those who responded to the invitation received by e-mail or telephone were included. A total of 19 subjects were enrolled among those who agreed to receive an objective examination of the oral cavity along with an analysis of the subgingival plaque. The plaque was collected with sterile endodontic paper points placed in the gingival sulcus for 30 s. The DNA was extracted from the cones in two successive phases. Conclusions drawn from the study highlighted that there is no hypervariable region that has the same diagnostic power for all the bacterial genera examined; therefore it would be advisable to use different regions in a combined manner. One example is found in *Synergistetes* that on the one hand are not identifiable by the primer for V5–V6 and on the other, if searched with the primer V1–V2, they represent more than 1% of the classes. The outcome was similar for *Fusobacteria*. *Clostridia* instead are four times more abundant in the samples analyzed with V5–V6 [12].

Metagenomics has been demonstrated to have some limitations. In fact, even if high-throughput sequencing methods have dramatically reduced the time of analysis of samples owing to integrative workflows, Lagier et al. explained that metagenomics cannot discriminate between live and dead bacteria and that they need at least bacteria with a concentration of 105 cells per gram to detect a population [41].

Studies in literature are not homogeneous because the choice of the primer used for the hypervariable region of the 16S analysis and the platform selected for metagenomics can greatly modify the results [41]. However, it is interesting to note that, despite the previous studies in which the subgingival and peri-implant flora seemed to be quite similar, in the new ones that use the metagenomic approach such results seem not to be confirmed.

The majority of studies in literature use paper points for sampling. As has emerged from the studies by Van der Horst et al. and Pèrez-Chaparro et al., paper points are the main source of bacterial contamination, and the adsorbent properties of the points make very easy to collect the crevicular fluid (with desquamated cells, lysed leukocytes and human DNA debris) rather than bacteria. Therefore, authors suggest collecting sub-gingival plaque samples with sterile curettes [42,43,44].

Illumina (Miseq) allows us to obtain rapidly profiles of relevant high-resolution taxonomic abundance of microbial communities; in comparison to pyrosequencing, it guarantees a greater depth of sequencing, reduced costs and a smaller number of errors. The flip side of the coin is that, by producing short sequences, it does not make the taxonomic assignment simple [12].

In the systematic review of relevant literature of Padial-Molina et al. the various techniques with which the peri-implant microbiota was studied were examined: The culture, the DNA–DNA checkerboard hybridization technique, the PCR and the 16S rRNA [44]. The 16S analysis hardly distinguishes differences at taxonomic levels lower than the genus; furthermore it is impossible with the 16S rRNA analysis to outline the pathogenicity of an organism and its virulence factors [12,44]. It is also impossible to distinguish DNA from transient bacteria, nor to discriminate live bacteria from dead ones [45,46].

Many authors, in light of the International standards for genomes, transcriptomes and metagenomes affirm, moreover, that the 16S rRNA analysis (even when using multiple regions) is one of the least effective genes for distinguishing closely related species, and it is not even the best gene for distinguishing distantly related species [47,48,49]. Another limitation of metagenomics is the challenge of understanding how much DNA is necessary to obtain a correct analysis [50].

Starting from the provocative question of Ruppè et al.: “since metagenomic sequencing techniques can detect bacteria (or viruses) never identified before, these should be considered false positives?” [51], all of the scientific community began to consider that the use of metagenomics to identify new pathogens is a difficult challenge since any new pathogenic suspect identified with metagenomics must be confirmed as a minimum in a group of subjects and through the use of other approaches considered the gold standard, like the culture [51].

Despite all the highlighted limitations of metagenomic and 16S gene analysis approaches such techniques contributed in clarifying some crucial aspects of the oral microbiome. Basing on the reported evidence some bacteria like *Fusobacterium nucleatum*, *Prevotella intermedia*, *Porphyromonas gingivalis*, *Aggregatibacter actinomycetemcomitans* and *Filifactor alocis* can be found around teeth and implants even in the absence of signs of inflammation and *Actinomyces, Neisseria* and *Rothia* spp. are more frequently found in supragingival peri-implant biofilm. Teeth and implants (even if adjacent) seem not to share the same microbiome and healthy teeth have a more diversified one. Metagenomic and 16S gene analysis also highlighted that the oral biofilm of smokers is composed of more periodontopathogen bacteria compared to non-smokers and that geographical location and ethnicity seem to play a role in bacterial composition.

With the aim of compensating the limits of the molecular methods a new cultural analytic approach was developed: Culturomics. 

### 4.3. Future Perspectives: Culturomics

Due to metagenomics, molecular techniques have supplanted cultural methods, which are characterized by longer analysis times and a high degree of laboriousness. However, some studies have suggested that molecular investigation techniques covered a different spectrum than the one investigated with cultural methods [52,53]. Lagier et al. demonstrated the complementarity between culture-dependent and culture-independent methods since the two techniques succeeded in identifying concomitantly only 15% of species. This discrepancy in the identification of microorganisms with the two approaches induced researchers to carry out larger culture-based studies to complete the characterization of the microbiota of the human intestine [54].

Culturomics consists of the use of different culture conditions and of the identification by matrix-assisted laser desorption ionization–time of flight mass spectrometry (MALDI–TOF MS) with the aim of increasing the bacterial repertoire [54,55].

The first aim of culturomics was to provide different culture conditions with the aim of promoting the growth of fastidious bacteria living in the human gut [55]. Culture media were improved using blood and rumen fluid in blood culture bottles and the growth of minority populations was promoted [41]. In the first reported culturomics study, 212 different culture conditions generated more than 30,000 colonies. Among all the bacterial species isolated 31 were new or belonged to rare phyla [41,54].

Culturomics consists of different laboratory phases. The first step is the division of the sample and the establishment of different culture conditions [55]. Such conditions suppress the culture of majority populations and improve the growth of fastidious microorganisms. Culturomics allows the identification of bacterial species by MALDI–TOF mass spectrometry in less than one hour. If identification fails, colonies are analyzed through the 16S ribosomal RNA (rRNA) sequencing. The identification of new species by culturomics allowed us to increase the repertoire of bacterial species associated with humans. Moreover, culturomics allows for obtaining living bacteria that could be investigated for different microbiological features; for example, each isolated colony could be tested for antimicrobial susceptibility. This is a very important aspect concerning the safety and identification of a resistant bacterial strain [41]. As regards in describing microbiota there are mainly two strategies: Metagenomics, which has highlighted the gut microbiota diversity but also revealed that the majority of bacteria in the gut remain uncultured, and culturomics that was developed to culture and identify previously un-identified bacteria [55]. For all these reasons culturomics provided exciting new perspectives on host-bacteria relationships [54,56,57,58,59,60,61,62,63]. Metagenomics in addition to undeniable positive aspects such as analytical quickness, multiple sample analysis and detection of taxa relative abundance, presents many limitations such as the possible bias during DNA extraction protocol, a limited depth of analysis (bacteria population under 105 cells per gram are undetectable) and the inability to discriminate between live and dead bacteria. The culturomic method despite the long analytical times has a greater analytical depth allowing the detection of bacterial populations up to concentrations of 102 cells per gram [53]. An important limitation of culturomics, however, must be highlighted: Being an approach based on the induction to the growth of the greatest number of bacterial species present on the sample it is evident that the analytical result can only be qualitative. No quantitative information can be drawn. This negative aspect, however, can be avoided by combining culturomics with the real time PCR that, however, obviously causes an increase in costs.

By adopting culturomics in dentistry, indeed, it will be possible to expand the knowledge regarding oral health and pathological conditions afflicting this district beyond the current limits. In order to better evaluate perspectives and limits of the all presented approaches further studies comparing the different molecular techniques are encouraged.

It is necessary to underline an important limitation of the present review. The absolute lack of articles comparing the metagenomic, culturomic and 16S gene analyses in the study of the periodontal and peri-implant microbiota composition led the authors to the impossibility to satisfy the primary aim of the review. It would be desirable that, in the future, other authors could investigate these three methods and comparing them in order to provide information that can be analyzed with a systematic approach and give a greater specific weight to scientific knowledge.
